# Perception of Dating Violence in Teenage Couples: A Cross Validation Study in Spain and Colombia

**DOI:** 10.3390/ijerph17186769

**Published:** 2020-09-17

**Authors:** Ana Martínez-Dorado, Jesús Privado, Sergio A. Useche, Lilian Velasco, Dau García-Dauder, Elisa Alfaro

**Affiliations:** 1 Department of Psychology, Rey Juan Carlos University, Escuela Internacional de Doctorado, 28922 Alcorcon, Spain; a.martinezdor@alumnos.urjc.es (A.M.-D.); lilian.velasco@urjc.es (L.V.); dau.dauder@urjc.es (D.G.-D.); 2 Faculty of Psychology, University Cardenal Cisneros, 28006 Madrid, Spain; jesusprivado@universidadcisneros.es; 3 Department of Psychobiology and Methodology in Behavioral Sciences, Faculty of Psychology, Complutense University of Madrid, 28223 Pozuelo de Alarcon, Spain; 4 Faculty of Psychology, University of Valencia, 46022 Valencia, Spain; m.elisa.alfaro@uv.es

**Keywords:** dating violence, risk profiles, sexism, adolescence

## Abstract

Background: dating violence, or violence in teenage couples, is a socially interesting topic, due to its prevalence and its possible use in predicting violence in adult couples. The perception of violence, or the detection of abusive behaviors by teenagers and young people (which can be considered as equivalent concepts), is essential to prevent violence itself. Therefore, the main objective of this research is to determine which behaviors are identified as abusive by teenagers and young people, and the severity that they attribute to them—meaning how they perceive them. Moreover, we will be able to determine whether there are differences between boys and girls in two countries: Spain and Colombia. Methods: for this study, we used two samples from both countries, with a total of 389 teenagers (50.9% females and 49.1% males) who were, on average, 16.56 years old (SD = 1.94 years). We analyzed the factorial invariance depending on sex and country of the sample and the different profiles of violence perception. Results: we found evidence of the internal validity of the questionnaire for what concerns the perception of inter-partner violence. The results point out that the perception of violence in the relationship is composed of two factors related to each other (Multiple and Emotional Abuse), which are invariant depending of sex and country of origin of the sample. The internal consistency of the test is adequate (>0.90). The analysis of the violence perception profiles indicates that Spanish teenagers have a higher perception of it, and, also, that girls hold a higher perception than boys. Conclusions: the results of this research have shown how dating violence (or violence in teenage couples) is differentially perceived not only between genders, but also across cultural contexts. Moreover, these outcomes may enhance the development of possible evidence-based interventions approaching the social problem generated by violence in teenage couples.

## 1. Introduction

The study of violence in teenage and young couples, also called dating violence, has attracted much attention in the past few years, especially because of its high incidence. There are multiple studies that determine the bidirectionality of violence between the members of a couple; however, there are others that determine existing asymmetry in couples, taking into account the gender perspective. Therefore, they emphasize the role of violence against women within the couple (*Intimate Partner Violence Against Women*, or IPVAW), understanding this concept as violence exerted by a man on a woman who is, or used to be, his intimate partner [1].

Specifically, violence against women is one of the main worldwide issues faced by public health. The United Nations Organization (UNO) estimates that 35% of women, on a global level, have suffered some type of physical and sexual violence during their lifetime, and this number does not consider data on violence perpetrated by their partners [1]. Moreover, around 7% of women have suffered sexual assaults from someone who is not their partner [2].

Violence against women (or gender violence) is not a biological nor a domestic issue, but rather a gender-related one. Gender is the results of a social construction process, through which expectations and values are symbolically imposed on both sexes; each culture holds its own expectations towards men and women. This theoretical value is essential to understand that the reason for the antagonism is not the difference between sexes; indeed, we are not facing a form of individual violence that is exerted within the family, or couple, by someone who presents physical superiority over the weaker sex, but rather a consequence of historical discrimination that has its origins in a patriarchal social structure, [3].

As we initially described the majority of studies on dating violence do not take into account gender perspective. This has an influence on how the violence exerted is assumed bidirectional, not only because of conceptual issues, but also because of the type of instruments used to measure it [4]. On the other hand, studies with a gender perspective provide percentages and data on the severity of violence against women exerted by their partners or ex-partners that contradict the bidirectionality and symmetry reflected in studies that researched dating violence.

Even though dating violence is a much less studied phenomenon in comparison with violence against a partner in adults (also called intimate violence within the couple) or gender violence, during the past few years different definitions have been suggested. Such definitions do not have universal consensus, but they do highlight some common elements: (1) (intentional) threat or provocation of a real damage, either physical, sexual or psychological; (2) control or dominance over the partner (through threats, or coactive/coercive tactics); and (3) that threats, coactions, control, domination, or damage are produced within a dating relationship. There is scientific unanimity, when referring to “dating relationships”; we are referring to teenage or young people, while relationships of single, separated, widowed, or divorced adults remain on the sidelines [5]. Therefore, when inter-partner violence occurs in teenage (or young people’s) relationships, it has been named “dating violence”. Conceptually, dating violence refers to every type of violence, physical, sexual, and psychological, perpetrated during a relationship [6].

With regards to the nature and structure of dating violence, there are various tendencies indicating that there is no difference with intimate couple violence, since they share risk and precipitating factors, such as, for instance, alcohol abuse, a deficit in communication abilities, or a history of intra-familiar violence. However, they do have some differentiating elements, such as (1) dating violence does not always lead to assaults within the couple after marriage or after initiating a co-habiting situation, and (2) not every abuser within adult couples has displayed aggressive behaviors during the dating stage. Moreover, relationships in married couples are characterized by the presence of a family unit with a common economy, and by the frequent presence of children, which are generally absent in the dating relationships of teenagers and young people. On the other hand, factors such as peer pressure, as well as a higher relevance of gender roles and dominant behaviors of males towards females, are more present in teenagers and young people [7].

### 1.1. Psychosocial Consequences of Violence within the Relationship

Because intimate partner violence and dating violence share some common factors, we will highlight some of the psychosocial consequences left by violence in an affective relationship. As unpleasant behaviors, such as control and jealousy, begin to take place within the couple, the relationship starts to be affected and this generates an impact on the bond between the two members, causing deep discomfort, and leading, in some extreme cases, to violent endpoints. Inter-partner violence is a multi-causal phenomenon that causes severe physical, psychological, social, and economic consequences in its victims [8,9,10]. Concerning the psychological consequences, depression, anxiety, suicide, sleep disorders, bad mental health perception, and post-traumatic stress disorders stand out; regarding physical consequences, we have pain, fatigue, muscular and bone pain, cardiovascular diseases and diabetes, and respiratory and gastrointestinal diseases [11]. Violence, when directed towards women and related to a gender situation, is a mean of strengthening the dominance of men over women [12,13], causing the victim to experience clinically significant discomfort [14].

Women, who suffer the most severe consequences of inter-couple violence, are especially penalized when this violence is physical; this does not however imply that men cannot be victim of inter-couple violence, but this is not ‘gender violence’ or a structural problem [8,15,16]. The majority of victims of this kind of violence are women, especially under 25 years old [17]. In the European Union, one out of every ten women says she has been suffering sexual or psychological cyberbullying since the age of 15. Harassment of women increases when the victims are between 18 and 29 years old. There is also evidence of violence and abuse of women with a different sexual orientation, women with disabilities, and transsexual women [18].

In one of the existing studies, it was observed that 15.1% of women and 26.5% of men had reported some form of physical victimization, and, with regards to physical injuries, 15.9% of women and 6.9% of men reported at least one injury [19]. In addition, research on female perpetrations has been increasing, while, on the other hand, studies on male victims are fewer [20]. However, this kind of study does not take into account power differences, sexual violence, or symbolic violence, as it only looks at the behavior data out of context.

Different studies have found that inter-couple violence can cause traumatic and psychopathological reactions in victims, such as post-traumatic stress disorder, depressive symptoms, and low self-esteem, with a higher prevalence in young victims who have experience forced sexual intercourse [21,22].

### 1.2. Dating Violence and Perceived Violence

Now focusing on dating violence, the evidence tells us that it can predict future violence in adult couples. The type of aggressions that happen in dating violence can cause physical, sexual, psychological, and emotional wounds in those who experience it [23]. The most common type of violence is psychological, followed by that of a physical and sexual nature [24].

In other studies, it was found that victims of this type of violence can adopt risky sexual behaviors (such as not using condoms), unhealthy eating habits, use of substances, such as alcohol, and even display suicidal behaviors, as psychological consequences of the aggression [25].

Additionally, one of the problems generated by dating violence is rooted in not being perceived as violence by the victim themselves. We understand the perception of violence as the detection (or lack of it) of violence, as well as the intensity or frequency with which it is detected [23]. It has been found that behaviors associated with dating violence are not necessarily considered to be abusive by teenage women, therefore being downplayed and, consequently, not reported. This lack of violence perception is due to cultural factors framed within gender violence [1]. In addition, such factors can normalize violence and its associated behaviors, thus, fostering the generation of situations of normality and sexist culture [26,27]. According to Urbiola et al. [28], those who are better able to detect violence are young women between 25 and 30 years old who have never been in a relationship. On the other hand, violence is worst perceived when the person is male, has been in at least one relationship, is between 16 and 17 years old, and has experienced at least one violent situation of any type.

It was found that women victims of violence present a worse psychosocial profile and worse communication with their relatives, which can facilitate their permanence in these violent relationships. Other variables related to the perception of violence are the concepts of family held by those who experience abuse, and, therefore, a lack of adequate communication with families can turn into a factor of victimization in teenage women [29]. This would indicate a normalization of violence, so that their perception of certain behaviors will be perceived as less severe.

Within the perception of dating violence, [30], there is a distinction between two types of abuse: (1) multiple, which includes situations in which: pictures of the victim or insults directed at him/her are spread on the internet or through cellphones without his/her consent; he/she receives threatening messages, or is physically threatened so that he/she will do things he/she does not want to do; he/she is pressured into sexual acts, physically assaulted, verbally intimidated, sexually abused and blamed for the violence he/she suffers. (2) Emotional, which includes situations in which: he/she is subjected to abusive control, isolation from his/her friends, he/she experiences fear and behaviors that lower his/her self-esteem. Emotional abuse appears in the first stages of dating violence, and it is more frequent and prevalent if compared with multiple abuse. Emotional abuse is related to psychological aspects, in turn associated with control, differently from multiple abuse, which includes other behaviors that imply threats or (intentional) provocation of a real damage, either physical, sexual or coactive. Teenage girls tend to assess these behaviors as more serious than boys.

There is a series of intrapersonal factors and situations that propitiate this type of violence, with a series of variables that increase the risk of victimization, related to strategies of confrontation and distancing, as well as other variables that increase the risk of aggression, such as anger, hostility, jealousy, controlling behaviors, and antisocial conduct; factors associated with dating violence in teenagers [5].

### 1.3. Measuring the Perception of Dating Violence in Teenage Couples

As mentioned at the beginning, there are different perspectives in the assessment of dating violence. On the one hand, there are those that must include gender violence, due to its incidence and to the severity of this problem on a social level. There is a study that constructed a test using the following elements as indicators of gender violence: harassment, isolation, jealousy, discrediting, emotional indifference, sexual pressure, and emotional manipulation. Two components consistent with the gender violence theory were identified: (1) hostile dominance (threatening behavior and sexual pressure) and (2) controlling-passive dominance (jealousy, control over the other person’s time, possessions, and social relationships with other people). Negative correlations were found between the capacity to spot gender violence and hostile sexism (−0.569), benevolent sexism (−0.469) and the psychological abuse received (−0.451) [4]. 

Concerning the most recent instruments used to measure the perception of violent behaviors in teenagers, we have the Gender Violence Questionnaire 2.0 [15]. This questionnaire gathers information on the perception of the internet as a violent world that holds no accountability, as well as on having vulnerable personal characteristics, normality of violence between sexes, experiences suffered in a virtual environment, and ways of behaving when experiencing this violence. The internal consistency data are quite high (between 0.901 and 0.961, according to the analyzed subscale), and they present evidence of content validity [15]. In addition, they show that violence is essentially masculine: the perception of violent behaviors is more related to gender stereotypes, to sexual violence and to violence as a consequence of manifesting anti-patriarchal positions.

However, there are other studies that use measurements claiming that dating violence is a phenomenon with more than one determined form: there are situations that follow a unidirectional path, and situations where the path is more similar to the symmetry [31,32]. They found that teenage women tend to manifest a higher use of aggressive psychological and mild physical tactics, compared to men. Likewise, other studies reveal that a higher percentage of women use verbal violence (95.3% vs. 92.8%), while men commit more severe physical violence (4.6% vs. 2%) [31].

Rey-Anacona developed a list of abusive experiences suffered within couples in Colombia, in which 95 abusive behaviors towards the partner are described: physical, verbal, emotional, psychological, sexual, economic, and neglect-related [16,33]. The questionnaire presents evidence of content validity, granted by judges specialized in intra-familiar violence, and an internal consistency of 0.96 [16].

Díaz-Aguado [30] developed a questionnaire that gathers 14 behaviors related to the perception of dating violence in teenage couples. They validated the questionnaire with a representative Spanish sample of more than 11,000 participants between 14 and 24 years old. The internal consistency of the test was 0.953. Regarding evidence of the internal validity, they obtained that when the questionnaire was applied, asking about the perception of abuse from a boy to girl, all 14 behaviors could be summed up in two components (multiple and emotional abuse), which explained 68.7% of the total variance. However, these two components presented a correlation that was quite high (*r* = 0.709), which could suggest a second-degree order that may explain them. On the contrary, when the questionnaire was applied asking about the perception of abuse from a girl to a boy, there was only one general component that summarized the information and explained 63.7% of the variance. Following [28], it is advisable to employ maximum likelihood extraction processes when attempting to obtain evidence of a test’s internal validity, instead of principal components as [30] did, since this latter procedure does not allow for obtaining latent factors. Afterwards, [23] obtained evidence of the test’s internal validity by using an Exploratory Factorial Analysis, in which they found a latent factor and a 0.95 internal consistency. This is the questionnaire used in the present research. 

### 1.4. Dating Violence Situation in Spain and Colombia 

In Spain, not only the existence of dating violence was found, but also its normalization [24]. Díaz-Aguado [30] found that 10.9% of women said they had suffered some type of violence perpetrated by their partners, and 3% of them said it had happened during the previous year. If we focus on women between 18 and 29 years old, percentages increase to 12.3% and 3.7%, respectively. Moreover, rejection of sexism does not have the same proportion in both sexes, since women perceive violent behaviors as more serious. It also points out that during 2018 there were 49 casualties due to gender violence in Spain. Up to 249 minors were prosecuted for sexist violence in 2018, and measures were taken in 92.37% of cases. Requests of protection measures by underage girls also increased from 963 to 1010. 

In Colombia, we have fewer studies on this type of violence. In 2005, out of 704, 34 reported cases of domestic violence, 25.43% corresponded to people between 18 and 24 years old, and 2.51% to individuals between 15 and 17 years old [34]. Rey-Anacona [16] informs that there is an 82.2% prevalence of violence in single teenagers and youngsters; distinguishing between the types of violence, we would have 22.4% prevalence of physical violence, 81.1% of psychological violence, 31.5% of emotional violence, 8.3% for sexual violence, and 18.2% for economic violence. In the cases of sexual and economic violence men exerted violence more than women. During 2018, the National Institute of Legal Medicine and Forensic Sciences of Colombia performed 46,669 assessments in the context of inter-partner violence, 86.08% (42,753) of which corresponded to women, with Bogotá, Antioquia, Cundinamarca, and Valle del Cauca being the departments with more allegations. Moreover, 49.24% of cases (24,456) occurred in couples from the young or teenage population (between 10 and 29 years old). Out of all cases of violence against partners reported, 86.08% corresponded to violence against women [34].

### 1.5. Spain and Colombia: Cultural Focus and Dating Violence 

Regarding existing studies on sexist attitudes and their relationship with intimate partner violence, if we talk about Ibero-America, only studies developed in Spain and Colombia are available, and not many of them [35]. One of these studies shows results coherent with the statement that sexism has a differential weight on violence, depending on the type of violence that is analyzed. This leads us to conclude that, future research must compare the differential weight of sexist attitudes depending on their culture of provenance, and on the level of generalization of the findings in relation to the studied phenomenon [36]. 

Regarding this, Spain and Colombia share the same language and cultural patterns, but they present very different indexes of gender inequality (Gender Inequality Index, GII), which suggests very uneven human development indices too [37]. Even though sexism exists in many societies, its presence in Latin America is higher, since teenagers show remarkable dominance, power exertion, aggressiveness, hypersexuality, and leadership as socially normalized behaviors within couples [38].

The scarce efficiency of norms’ implementation, the existence of very low allegations indexes, the lack of a comprehensive perspective on gender violence and the normalization of inequalities prevent the actions on gender equality taken in Latin American countries from being successful [39,40]. 

### 1.6. Objectives and Hypothesis of the Study

Intimate partner violence is an interesting social issue, since it presents some risk factors that increase its probability to occur: hostility, jealousy, and controlling behavior. Therein lies the interest of measuring young people´s perceptions of this type of violence. 

Therefore, the main objective of present study is to determine which behaviors are perceived as abuse by teenagers and young people, as well as their severity—meaning how they perceive them. Moreover, we will be able to determine whether there are differences between boys and girls in two countries: Spain and Colombia. First of all, we validated the questionnaire of inter-partner violence by Díaz-Aguado [30] in the two samples, in order to verify whether the factorial structure was equal to the one proposed by the authors of the scale in both countries and in both sexes. In this case, we employed a confirmatory process. Secondly, we studied whether there were differences depending on sex and country in the different factors measured by the questionnaire and its items, with the purpose of finding out which profiles could be found depending on the aforementioned measures.

With regards to the questionnaire´s internal structure, we expected to find, as it happened to the authors of the test [30], two factors correlated to abuse (multiple and emotional) in the case of boy-to-girl violence. However, only one factor was expected to be found for what concerns the perception of girl-to-boy violence. We did not expect to find differences in the factorial structure (number of factors) in both sexes and in the samples’ countries (Colombia and Spain).

Moreover, based on previous research and on the fact that women are better at perceiving abusive behaviors when they are the victims [28], it was reasonable to expect that violence would be more perceived by girls than by boys when the latter are the perpetrators, but not the other way around. In addition, it was expected that the perception of violence would be higher in Spain than in Colombia, since gender values and stereotypes are much more rooted in Latin America, thus making them more difficult to detect, and complicating the task of attributing them the same importance that they are given in Spain.

## 2. Materials and Methods

### 2.1. Sample

The full study sample was composed of 389 teenagers with a mean age of 16.56 years old (SD = 1.94 years), 30.1% of which were Colombian, while the rest were Spanish; 50.9% of them were women.

The Spanish sample was composed of 272 students from the 3rd and 4th years of middle school and from high school, from different autonomous communities, and had an average age of 16.92 years (SD = 1.78 years); 51.1% of them were women.

The Colombian sample was composed of 117 students from middle school, belonging to public and private centers, attending 1st and 2nd years, with an average age of 15.72 years (SD = 2.4 years); 50.4% of them were females.

### 2.2. Measures

For this study, the *Questionnaire of Perception of Inter-Partner Violence* was used, created by Díaz-Aguado [31]. It is composed of 14 violent behaviors displayed or perceived by teenagers, and validated with a representative Spanish sample. It is a questionnaire answered using a Likert scale format with 4 options: a lot, quite a lot, not really, not at all. Both boys and girls responded on the perception they had of violence perpetrated by boys towards girls, and by girls towards boys. In order to do this, they completed the questionnaire twice. The employed items are shown in Table 1 and Table 2.

We have defined violence perception as the detection of abusive behaviors, and the assessment of each of these behaviors within the following scale: none, a little bit, quite, a lot. The “none” answer implies the lack of perception of these behaviors as abuse. However, the three remaining answers (none, a little bit, quite, a lot) imply the existence of this perception, as well as a shade of intensity in the behavior (its severity). In addition to allowing us to detect the behaviors themselves, it also allows for the detection of the ways these abusive behaviors appear (multiple and emotional abuse).

The obtained sociodemographic data were mainly age and sex. Other data were collected, but they are not relevant for the research. Having experienced an affective relationship was not established as a requirement to answer the questions, and, therefore, it was not asked. The instruction was for participants to answer whether they considered the presented behaviors as not abusive (therefore answering “none”) or, if they did consider them as such, how severe they perceived them to be (none, a little bit, quite, a lot).

### 2.3. Design and Procedure 

The questionnaires were applied online through Google Drive for both samples. Participants were invited to take part in the study through the intermediation of their educational centers. As for the sampling technique, a convenience (non-probabilistic) method was employed, founded on the accessibility to the study population and their willingness to participate in the study. All participants were initially informed about the importance of answering honestly to all questions, as well as about the non-existence of wrong or right answers. The exact instructions given were the following: “*this questionnaire is anonymous: therefore, it will not be associated with any names. You will now be presented with a survey, that you must answer honestly. There are no correct or incorrect answers. The objective is to determine whether the presented behaviors represent abuse, into your opinion. You have four options for your answers: NONE (if you think that the behavior does not constitute abuse), A LITTLE BIT (if you think that the behavior is an abuse, but not a severe one), QUITE (if you think that the behavior is abuse, and quite a severe one), A LOT (if you think that the behavior is extremely abusive)*”. Moreover, and since the on-line questionnaire was applied in Spanish, it was translated, adapted, and re-translated by qualified professionals. Before applying the test in Colombia, a linguistic adaptation was performed by a group of five students and a professor from the Universidad Javeriana (Bogotá, Colombia). The study was approved by the Ethical Committee for Studies with Human Subjects, and all participants gave their consent before taking part in the research.

### 2.4. Statistical Analysis

First of all, we carefully curated the data, calculated the study variables, and analyzed the distribution of the different items of the questionnaire, in order to find out whether the distribution was normal. Specifically, normality was calculated by dividing the skewness and kurtosis indexes by their corresponding errors. If these values are not greater than |1.96|, normal distribution will be assumed. Secondly, we analyzed the evidence of the questionnaire’s internal structure by means of the AMOS Version 17.0 software (IBM, Armonk, NY, USA, 2013) [41]. This analysis requires at least 3 indicators for each latent factor, with a minimum of 100 participants and 10 times the number of observed variables [42]. In the Spanish sample, these criteria are fulfilled with 272/14 ≈ 19 participants per indicator; in the Colombian sample, the only criterion that could not be fulfilled was the number of participants per indicator (117/14 ≈ 8), even though the value is close to the ideal one. The procedure employed to adjust the models was generalized least squares, since it is the appropriate procedure when the variables are ordinal [42].

The goodness of fit statistics used to evaluate the adequacy of the models were: (1) absolute fit of the model to empirical data with the statistic χ^2^. With the null hypothesis, the matrix theoretical and empirical data being equal, this is commonly rejected with large samples, so the ratio χ^2^/df is often used [41], indicating a good fit with values lower than 3. Another absolute fit index is Root Mean Square Error of Approximation (RMSEA) [43] whose values below 0.05 indicate good fit, and Standardized Root Mean-Square (SRMS) [44], or below 0.08, according to other authors [45]. Moreover, another absolute fit index that needs to be examined is the standardized residuals matrix: in the case of having few values below |1.96|, we can consider that there is a low discrepancy between the covariance matrix observed and estimated for the model. (2) Incremental fit measures compare the resulting model with the null model. Normed Fit Index (NFI) [46], Tucker Lewis Index (TLI) [47], Goodness of Fit Index (GFI) and Comparative Fit Index (CFI) [48] are the most frequently used. Values above 0.95 indicate good fit and point that the empirical model is significantly different from the null model. (3) Parsimony fit measures evaluate the model fit versus the estimated number, taking into account the complexity of the hypothesized model in the assessment of overall model fit. Parsimony Goodness of Fit Index (PGFI) [49] and Parsimony Normed Fit Index (PNFI) [50] are more representative coefficients, and values above 0.50 indicate good fit. Third, once we obtained the definitive factorial structure, the reliability (internal consistency) of the questionnaire was calculated through Cronbach’s alpha, considering a suitable value at least 0.80. In addition, the point-biserial correlation of each item with the total of the scale was calculated to see the internal discrimination of each item.

Thirdly, we analyzed the factorial invariance depending on sex and country (Spain or Colombia), by means of the AMOS Version 17.0 software. The invariance was calculated for each version of the scale separately, that is, for the boy-to-girl version and for the girl-to-boy version, separately. It was checked whether there was metric, strong metric and strict metric invariance in the confirmatory model obtained in step 2 for both sexes and countries. For this, the χ^2^ test values were not statistically significant at *p* < 0.05 level and the CFI increase does not go beyond 0.01 [51]. 

Finally, with the aim of assessing the profile factors of the questionnaire, we analyzed differences depending on gender and country of the participants through an ANOVA with independent factors; and, concerning the profiles of the questionnaire’s items. We interpreted the results based on statistical significance set at the *p* < 0.05 level, and also according to the effect size based on the *η^2^_partial_*. The size effect according to Cohen (1998)’s criteria is low for 0.01, medium for 0.06 and large for 0.14 [52]. Moreover, we used a logistic regression in order to see which ones explained the differences between each gender and country. We considered all the items together and we used a stepwise approach to select only those predictors giving a significant contribution to explaining the outcome. We use a criterion of *p* < 0.05 level to determine the relevant variables in the final equation. Statistical analyses were performed using the Statistical Package for Social Sciences (SPSS), version 24.0 (IBM, Armonk, NY, USA, 2017) [41].

## 3. Results

Descriptors. In Table 1 and Table 2 we observe the descriptors, asymmetry, and kurtosis indexes of the test’s 14 items, both for the boy-to-girl violence and for the girl-to-boy one. All of the items present negative asymmetry, which reflects that the perception of violence is quite high among the members of the sample, both boy-to-girl and girl-to-boy. Regarding the kurtosis, most of the items present a leptokurtic kurtosis.

**Figure 1:**
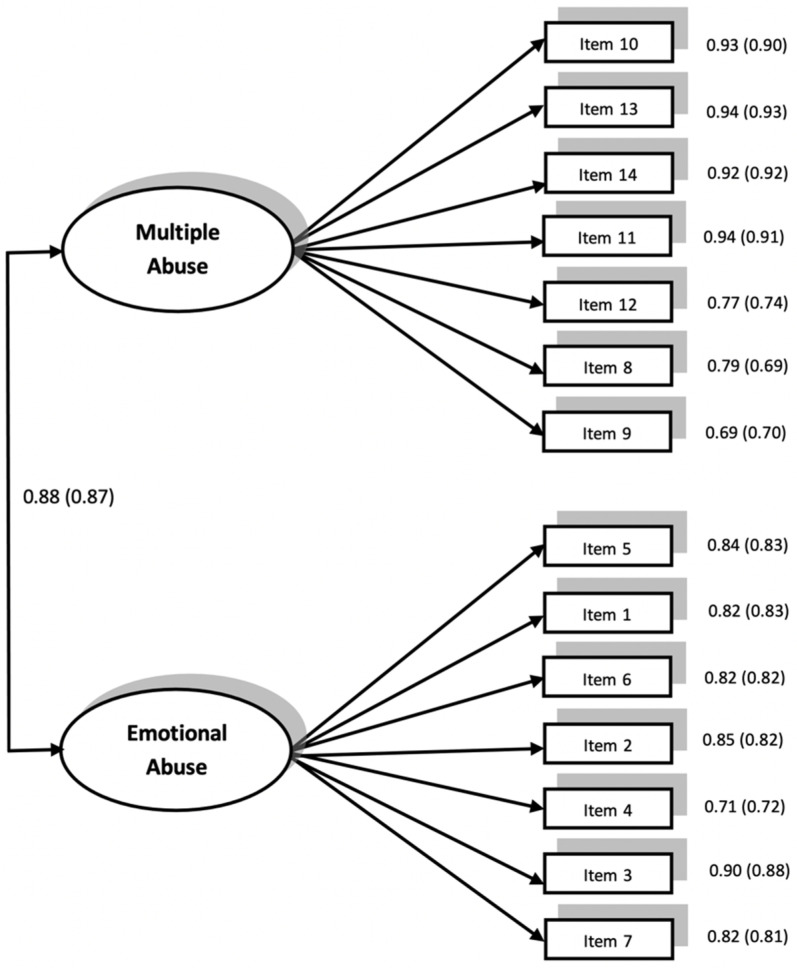
Confirmatory Factor Analysis of the Questionnaire of Perception of Inter-Partner Violence from boy-to-girl and girl-to-boy (in brackets). Factor loadings are standardized.

Evidence of internal validity. In Table 3, we see the goodness fit indexes for the two confirmatory models, which were contrasted for the two versions of the questionnaire in the whole sample: unifactorial and two factors related models. In both cases, it can be seen that the consideration of a two factors model provides a better fit than one latent factor alone. Moreover, the two factors model is well adjusted to the data. The two factors correspond to the two types of abuses proposed by Díaz-Aguado [30]: multiple and emotional. In addition, the two types of abuse present a quite high correlation, which implies that the perception of one of the two abuses leads to the perception of the other one as well. In Figure 1, we see the factorial weights of each item, above the two factors with quite high values (between 0.69 and 0.94).

Reliability. In Table 1 and Table 2, we see the correlations of each item with the total number of the factor to which they belong (multiple or emotional abuse) (biserial-punctual correlation). There are no items with correlations lower than 0.20; therefore, it would not be advisable to eliminate any of them [2]. The internal consistency (Cronbach’s Alpha) of the obtained factors was very high: (1) perception of boy-to-girl violence was 0.942 for multiple and 0.930 for emotional abuse; (2) perception of girl-to-boy violence was 0.930 for multiple and 0.926 for emotional abuse. 

Factorial invariance. We studied the factorial invariance of the questionnaire depending on the sex and country of participants. In Table 4 and Table 5, we have the results of the differences depending on, respectively, country, and sex. In both cases, when considering boy-to-girl violence the χ2 test is not statistically significant at 5%, and the CFI increase does not go beyond 0.01; we can therefore assume a strict metric invariance for both sex and country [45]. The presence of strict invariance implies that the factorial weights, the variance–covariance matrix, and the error variance are the same as the compared models. This means we can assume that both models are identical on a psychometric level. For what concerns the perception of girl-to-boy violence, the χ2 test is not significant at 5% level, and the CFI increase goes beyond 0.01 only when comparing C and D models related to the country of origin; we can thus assume a strong metric invariance, meaning that differences would occur only in groups compared by measurement errors. The presence of a strong invariance implies that the factorial weights and the variance–covariance matrix are the same, and the models are different only in what concerns the error variance, which in turn implies that the models, depending on the origin, are very similar to one another on a psychometric level. Therefore, we see that the factorial structure of the latent correlated factors is very similar in both versions of the test, depending on sex and country of participants.

Profile depending on sex and country. In Table 6, we see the ANOVA test’s results, using the two latent factors and with sex and country of participants as factors as well. Below, we will comment on those cases where statistically significant differences appeared. The assumption of homogeneity of variance is met in all analyses that were performed. First, for what concerns the country of participants, there are statistically significant differences in the emotional abuse factor in the perception of girl-to-boy violence, according to the origin of participants (*F*_1,385_ = 4.66, *p* = 0.031, *η^2^_partial_* = 0.012). The effect size is low (*η^2^_partial_*). The size effect according to Cohen (1998)’s criteria is low for 0.01, medium for 0.06 and large for 0.14 [52]. There is a higher perception of girl-to-boy violence in Spain (0.07) than in Colombia (−0.16). Second, for what concerns the sex factor, there are statistically significant differences in the multiple abuse factor (*F*_1,385_ = 6.40, *p* = 0.012, *η^2^_partial_* = 0.016) with low size effect. There is a higher average perception of violence in women (0.11) than in men (−0.11).

On the other hand, with the aim of establishing the country and gender profiles according to the test items´ answers, we conducted a logistic regression for each one of the variables. The two variables (gender and country) could not be combined, because the resulting sample size provided groups with only 58 participants. Therefore, the analyses were carried out separately for country and gender. Regarding country, the model of Boy-to-Girl violence classifies 70.2% of cases well, and manages to explain 11.7% of variance in the differences between countries (*R^2^ Nagelkerke* = 0.117); thus, presenting a good data fit (*χ^2^*(7) = 10.09, *p* = 0.184). In Table 7, the results of the contrast of the different predictors´ coefficients can be seen. All coefficients are statistically significant. The codification of the “country” variable was 1 for Spain and 0 for Colombia. According to this codification, since the model´s constant presents a positive value (*β*_0_ = 0.889), in absence of boy-to-girl abuse, Spanish teens have more odds in relation to Colombian ones. Their exponential value (e^0.889^ = 2.433) reflects that young Spaniards have 2.433 odds when no boy-to-girl abuse is perceived. For what concerns item 13 (Sending messages on the Internet or by cellphone which startle or threaten her), the positive coefficient (*β*_1_ = 0.596) indicates that young Spaniards have odds in the perception of this abuse. The exponential value (e^0.596^ = 1.816) indicates that Spanish teens perceive this type of abuse around 81.6% more than their Colombian counterparts. Regarding item 9 (telling him that if he leaves, she will hurt him), the coefficient is negative (*β*_2_ = −0.321), which indicates that young Colombians have odds in the item perception; that is, they perceive this issue 27.5% more than their Spanish counterpart. Moving on to item 7 (controlling everything that he does), the coefficient is negative (*β*_3_ = −0.880), which points out that Colombian teens have odds in the item perception, 58.5% more than Spanish ones. Finally, regarding item 2 (making her feel fear), the coefficient is positive (*β*_4_ = 0.481), meaning that young Spaniards have 61.8% odds of perceiving this abuse.

The model for girl-to-boy violence, taking into account the country, classifies 76.3% of cases well, and manages to explain 28.7% of the variance in the differences between countries [*R*^2^ *Nagelkerke* = 0.287], presenting a good data fit [*χ*^2^(7) = 5.28, *p* = 0.626]. All coefficients are statistically significant, except for the constant (see Table 7). In the case of items 4 (breaking something of his), 6 (preventing him from meeting his friends), 7 (controlling everything that he does) and 14 (disseminating messages, insults or pictures of him without his permission), the odds for the perception of these abuses have, in the case young Colombians, the following percentages of perception: 30.1% for item 4, 43.0% for item 6, 65.1% for item 7 and 41.8% for item 14. However, in the case of items 2 (making him feel fear), 5 (telling him who he can or cannot speak to or socialize with) and 12 (recording him on a cellphone or on video, or taking pictures of him without him knowing), the odds for Spanish teens, who perceive this abuse on a higher scale, are: 264.1% for item 2, 123.4% for item 5 and 92.6% for item 12.

For what concerns gender, the model for boy-to-girl violence classifies 58.6% of cases well, and it achieves the explanation of 4.6% of variance in the difference between genders [*R^2^ Nagelkerke* = 0.046], showing a good data fit [*χ^2^*(4) = 1.88, *p* = 0.758]. The codification of the gender variable was 1 for boys, and 0 for girls; therefore, positive values in the regression coefficients indicate odds for boys, and negative ones indicate odds for girls. In Table 7, it can be seen that gender differences are predicted in the case of two items only: 10 (hitting her) with boys having odds to perceive this abuse (47.7% more than girls), and 8 (insisting on having sex when she does not want to), with girls having odds to perceive this abuse (43.3% more than boys).

Finally, the model for the Girl-to-Boy violence according to gender classifies 63.0% of cases well, and manages to explain 7.1% of variance in the differences between genders [*R^2^ Nagelkerke* = 0.071], presenting a good data fit [*χ^2^*(6) = 9.22, *p* = 0.162]. In Table 7, we see the items predicting gender differences: item 8 (insisting on having sex when he does not want to), with odds for girls in the perception of this abuse (40.9% more than boys), and item 4 (breaking something of his), with odds for boys, with a perception 35.0% higher than girls.

## 4. Discussion

We will now answer the main objectives of this study, which are (1) to determine which behaviors are perceived as abuse by young people and teenagers, and (2) whether or not there are differences between girls and boys in two countries: Spain and Colombia.

In the present research, we obtained evidence of the validity and reliability of the inter-partner violence questionnaire, which had been previously published by Díaz-Aguado [30], in two samples of teenagers (Spanish and Colombian). Regarding internal validity, we obtained, using a confirmatory process, that the factorial structure of the questionnaire had a much better adjustment to the presence of factors, named multiple and emotional abuses, which present correlations between themselves. This structure is the same when we analyze the perception of boy-to-girl violence and vice versa. Previous studies had obtained only a two-factors structure in the case of boy-to-girl violence perception [30], while one factor was obtained in the case of girl-to-boy violence [24]. Moreover, we obtained that these two-related factors structure presented a metric invariance depending on both sex and country of origin of the sample, which gives the results a higher generalization. The reliability of the obtained factors, as a matter of internal consistency, was very high; therefore, we can state that they are a quite precise measurement of inter-partner violence.

Additionally, we studied the profiles in the two factors obtained through the questionnaire and the 14 items, according to sex and country of the sample, with the aim of finding out possible patterns in the perception of violence. Responding to one of the study’s objectives, the most remarkable results indicate that if we consider the country of origin of the sample alone, we find that Spanish teenagers perceive girl-to-boy violence more than their Colombian counterparts in what concerns emotional abuse. If we consider the results on an item level, we can observe that, when violence is produced by a boy towards a girl, young Spaniards have a higher perception of the behaviors of scaring her and sending messages on the internet or by cellphone, while Colombian teens have a higher perception of the behaviors of controlling what the other person does and threatening to attack her if she leaves. The first types of abuse (the ones that are perceived by Spaniards more) imply more straightforward actions than the other ones (perceived by Colombians more), which lean more towards controlling the other person, and are overall more implicit. On the other hand, when violence is exerted by a girl towards a boy, young Spaniards perceive scaring him, telling him whom he can or cannot speak to or where he can or cannot go, and recording him without his consent, as more frequent. Colombian teens perceive breaking something that belongs to him, preventing him from seeing his friends, controlling what he does and spreading messages without his consent as more frequent. Again, we find that the abuses that are perceived the most by Spaniards are straightforward actions, while Colombians perceive implicit and controlling actions as more frequent. Therefore, we clearly have a different profile for both countries in what concerns the perception of violence. Following this line, the fact that more physical or explicit violence is less perceived in Colombia is what could explain the data on violence against women within the couple that we have from this country, which is higher than in Spain [34]. The fact that Colombian teenagers’ perception of this violence is lower means there is a tendency to normalize it, and this could foster violent behaviors that are praised by culture [26,27,53].

Responding to the second objective, related to perception depending on sex, the pattern we obtained is that girls are the ones with the higher perception of violence in multiple abuse. These results coincide with previous data that considers women between 25 and 30 years old as the most capable of detecting violence [28]. If we analyze the information on an item level, gender differences point out that girls perceive the behavior of insisting on having sexual intercourse when the other person does not want to as more frequent, which could be influenced by the sexual education received by the two genders. However, in the case of boys, they tend to perceive the behavior of hitting the other person or breaking their things, which imply more physical violence of boys towards girls, as more frequent. This result matches previous studies, which indicate that boys perceive more violence in coercion and sexual pressure, while girls have a higher perception of psychological violence [54]. In any case, this data is relevant, since it shows that it would be adequate to pay attention to the differences in the perception of inter-partner violence depending on sex, as it seems that teenager girls perceive it more than boys.

Thinking of future research, it would be relevant to take into account some criterion-measurements that could not be considered in this study. For instance, it would be important to assess the relationships between the perception of violence within the couple and the measurements of psychic and physical discomfort, as it has previously been found by numerous studies [8,9,10,21,22,55]. Moreover, it would be interesting to study the relation between the perception of violence and some objective criteria, such as the number of physical or psychological aggressions that could be proven to have taken place. Likewise, it would be interesting to study how the perception of inter-partner violence occurs in non-heterosexual couples.

Finally, we should bear in mind the different systems in which the individual acts when measuring the perception of inter-partner violence. In order to achieve this, we could follow the ecological model for inter-couple violence [27], which assumes that the relationships in a person’s life are the results of interactions of various systems: the *ontosystem*, which refers to individual characteristics; the *microsystem* (which is the activities, personal relationships and role patterns experienced by the individual in his/her environment); the *mesosystem* (which is the interrelation of two or more systems); the *exosystem* (referring to environments that are not intervened by the individual, but can still influence his/her behavior); and the *chronosystem* (which represents the time changes in other environments), and the *macrosystem* (that is the broadest, and refers to the correspondences among micro-, meso- and exosystem at socio-cultural levels) [27]. For the case of this research, it was specifically at the *microsystem* where gender roles and/or stereotypes could be more clearly found, reflecting (e.g.,) role inequalities between men and women, highly gendered perceptions, and microsocial scenarios, potentially strengthening the likelihood dating violence. Furthermore, the model developed in this empirical study supports the assumption by which the interaction of these systems increases or diminishes the risk of dating violence; therefore, interventions should be focused on systems, rather than on the individual alone.

## 5. Limitations

The main limitations of the present study are the absence of evidence for the convergent and predictive validity of the validated questionnaire. Future research should try to approach this limited validity evidence. Additional measures, related to the perception of couple violence, should be collected. For instance, clinical symptoms, previous history of violence for one or both partners, time of permanence in the couple, violence suffered during childhood. Even the relationship between the scale and clinical personality factors could be analyzed. Likewise, it would be convenient to adopt criterion measurements that relate to the perception of violence within the couple: future issues in couples relationships, break-ups, behavioral problems within the couples, alcoholism problems, and psychological issues, such as stress, anxiety, or depression.

Another limitation is associated with the sample’s characteristics. These samples are not representative of the population, and they are limited to one age span only. Future research should try to replicate the data with additional samples, and to widen the age span of participants, with the objective of analyzing the study’s external validity. Moreover, another missing data is the socioeconomic status of the sample, which would be relevant for this type of study, benefiting potential income-based comparisons.

Finally, another limitation is that when measuring only the perception of behaviors out of context, the meaning, and importance of power relations is lost. It is necessary to investigate violence between young people, but in this case structural gender violence is only partially evaluated.

## 6. Conclusions

The findings of this study support the hypothesis that gender violence perceived among adolescents is higher in Spain and Colombia, and it is associated with demographic and relational factors. Furthermore, the existence of key sex-based differences (e.g., girls have a higher perception of dating violence) suggests the need to target and address the high-risk segments of the teenage population, in order to perform preventive and intervention actions focused on dating violence, as well as in further studies on gender violence among teenagers.

Moreover, the validated version of the *Questionnaire of Perception of Inter-Partner Violence* shows adequate fit, reliability, and consistency indexes, so it can be used for addressing teenagers’ dating gender violence in both countries.

This study endorses the value of evidence-based practices for the development of further studies in the field, with the aim of carrying out well-designed programs, systematic interventions, continuous evaluation, and improved approaches.

## Figures and Tables

**Table 1:** Descriptive statistics, asymmetry and kurtosis indices, and biserial-punctual correlation for each item from the different subscales of the Questionnaire of Perception of Inter-Partner Violence from boy-to-girl. Items from 1 to 7 measure emotional abuse, items from 8 to 14 measure multiple abuse.

Items	Mean	S.D.^1^	Standard Error	Skewness Index	Kurtosis Index	Point-Biserial Correlation	Alpha if Item Deleted
1. Telling her that she is worthless	2.01	1.02	0.05	−5.87	−2.55	0.788	0.919
2. Making her feel fear	2.24	0.96	0.05	−9.23	1.12	0.792	0.918
3. Insulting her	2.28	0.95	0.05	−9.94	2.02	0.836	0.914
4. Breaking something of hers	1.93	1.03	0.05	−4.57	−3.48	0.652	0.932
5. Telling her who she can or cannot speak to or socialize with	2.16	0.98	0.05	−7.97	−0.44	0.84	0.914
6. Preventing her from seeing her friends	2.2	1.01	0.05	−8.59	−0.27	0.808	0.917
7. Controlling everything that she does	2.07	0.96	0.05	−6.49	−1.14	0.736	0.924
8. Insisting on having sex when she does not want to	2.3	0.94	0.05	−10.12	2.27	0.77	0.937
9. Telling her that if she leaves him, she will hurt him	2.25	1.07	0.05	−8.97	−0.91	0.649	0.95
10. Hitting her	2.64	0.86	0.04	−19.39	17.99	0.874	0.928
11. Forcing her with threats to do things she does not want to do	2.52	0.87	0.04	−15.6	11.34	0.892	0.926
12. Recording her on a cellphone or on video, or taking pictures of her without her knowing	2.25	0.96	0.05	−9.19	0.92	0.756	0.938
13. Sending messages on the Internet or by cellphone which startle or threaten her	2.53	0.87	0.04	−16.01	12	0.891	0.926
14. Disseminating messages, insults or images of her without her permission	2.53	0.88	0.04	−15.57	10.69	0.885	0.927

Note: Item values are raw scores; ^1^ S.D. = Standard Deviation.

**Table 2:** Descriptive statistics, asymmetry and kurtosis indices, and biserial-punctual correlation for each item from the different subscales of the Questionnaire of Perception of Inter-Partner Violence from girl-to-boy. Items from 1 to 7 measure emotional abuse, items from 8 to 14 measure multiple abuse.

Items	Mean	S.D.	Standard Error	Skewness Index	Kurtosis Index	Point-Biserial Correlation	Alpha if Item Deleted
1. Telling him he is worthless	2.04	1.01	0.05	−5.82	−2.54	0.801	0.912
2. Making him feel fear	2.18	0.96	0.05	−8.01	−0.12	0.763	0.916
3. Insulting him	2.28	0.94	0.05	−9.62	1.74	0.799	0.912
4. Breaking something of his	1.95	1.03	0.05	−4.49	−3.57	0.663	0.926
5. Telling him who he can or cannot speak to or socialize with	2.12	0.98	0.05	−7.07	−1.23	0.841	0.908
6. Preventing him from meeting his friends	2.19	0.96	0.05	−8.42	0.34	0.817	0.91
7. Controlling everything that he does	2.1	0.98	0.05	−6.78	−1.4	0.698	0.922
8. Insisting on having sex when he does not want to	2.17	1.02	0.05	−7.99	−1.03	0.664	0.931
9. Telling him that if he leaves, she will hurt him	2.25	1.01	0.05	−9.02	−0.17	0.689	0.928
10. Hitting him	2.62	0.83	0.04	−18.53	17.16	0.833	0.914
11. Forcing him with threats to do things he does not want to do	2.49	0.88	0.04	−14.47	9.1	0.84	0.913
12. Recording him on a cellphone or on video, or take pictures of him without his knowing	2.29	0.96	0.05	−9.72	1.39	0.712	0.925
13. Sending messages on the Internet or by cellphone which startle or threaten him	2.5	0.85	0.04	−14.57	9.86	0.885	0.909
14. Disseminating messages, insults or pictures of him without his permission	2.52	0.87	0.04	−15.48	10.9	0.864	0.911

Note: Item values are raw scores.

**Table 3:** Goodness-of-fit for the contrasted models.

Perception of Boy-To-Girl Dating Violence											
Model	*χ^2^/df*	GFI ^1^	NFI ^2^	CFI ^3^	TLI ^4^	PGFI ^5^	PNFI ^6^	PCFI ^7^	RMSEA ^8^	SRMS ^9^	Standardized Residuals
											> |1.96|
Unifactorial	3.05	0.921	0.562	0.638	0.53	0.614	0.432	0.491	0.073	0.068	0.09%
Two related factors	1.87	0.953	0.735	0.849	0.801	0.626	0.558	0.644	0.047	0.033	1.90%
**Perception of Girl-To-Boy Dating Violence**											
Unifactorial	2.99	0.923	0.577	0.655	0.552	0.615	0.444	0.504	0.072	0.07	3.80%
Two related factors	1.68	0.957	0.765	0.883	0.864	0.629	0.58	0.67	0.042	0.03	1.90%

Notes: ^1^ GFI = Goodness of Fit Index; ^2^ NFI = Normed Fit Index; ^3^ CFI = Comparative Fit Index; ^4^ TLI = Tucker-Lewis Index; ^5^ PGFI = Parsimonious Goodness of Fit Index; ^6^ PGFI = Parsimonious Normed Fit Index; ^7^ PCFI = Parsimonious Comparative Fit Index ^8^ RMSEA = Root Mean Square Error of Approximation; ^9^ SRMS = Standardized Root Mean-Square.

**Table 4:** Goodness of fit indexes according to origin.

Specified Model	*χ* ^2^	*df*	*χ* ^2^ */df*	GFI	CFI	RMSEA	AIC ^1^
Model A. No restrictions (Unconstrained)	378.65	150	2.52	0.876	0.957	0.063	498.65
	*330.41*	*148*	*2.23*	*0.89*	*0.963*	0.056	*454.41*
Model B. Same factorial weights (Structural weights)	394.6	162	2.44	0.872	0.957	0.061	490.6
	*343.77*	*160*	*2.15*	*0.887*	*0.963*	*0.054*	*442.77*
Model C. Same factorial weights and variance–covariance matrix (Structural covariances)	420.9	165	2.55	0.869	0.952	0.063	510.9
	*352.48*	*163*	*2.16*	*0.886*	*0.961*	*0.055*	*446.48*
Model D. Same factorial weights, variance–covariance matrix and error variance (Measurement residuals)	457.07	180	2.54	0.854	0.948	0.063	517.07
	*439.41*	*179*	*2.46*	*0.854*	*0.947*	*0.061*	*501.42*
**Models’ Comparison**	**Δ*χ*^2^**	**Δ*df***	***p***		**ΔCFI**		
A and B models (metric invariance)	15.95	12	0.194		0		
	*13.36*	*12*	*0.343*		*0.002*		
B and C models (strong metric invariance)	26.3	3	<0.001		0.005		
	*8.71*	*3*	*0.033*		*0.002*		
C and D models (strict metric invariance)	36.17	15	0.002		0.004		
	*86.93*	*16*	*0.001*		*0.014*		

Notes: the perception of boy-to-girl violence appears in the first line, normal font. The girl-to-boy violence appears in the second line, in italics; ^1^ AIC = Akaike Information Criterion.

**Table 5:** Goodness of fit indexes according to sex.

Specified Model	*χ* ^2^	*df*	*χ*^2^/df	GFI	CFI	RMSEA	AIC
Model A. No restrictions (Unconstrained)	348.42	150	2.32	0.887	0.963	0.058	468.42
	*303.19*	*148*	*2.05*	*0.902*	*0.968*	*0.052*	*427.19*
Model B. Same factorial weights (Structural weights)	354.69	162	2.19	0.885	0.964	0.055	450.69
	*311.39*	*160*	*1.95*	*0.9*	*0.969*	*0.049*	*411.39*
Model C. Same factorial weights and variance–covariance matrix (Structural covariances)	358.32	165	2.17	0.884	0.964	0.055	448.32
	*321.5*	*163*	*1.97*	*0.897*	*0.967*	*0.05*	*415.5*
Model D. Same factorial weights, variance–covariance matrix and error variance (Measurement residuals)	413.02	180	2.3	0.869	0.956	0.058	473.02
	*420.68*	*179*	*2.35*	*0.873*	*0.95*	*0.059*	*482.68*
**Models’ Comparison**	**Δ*χ*^2^**	**Δ*df***	***p***		**ΔCFI**		
A and B models (metric invariance)	6.24	12	0.904		0.001		
	*8.21*	*12*	*0.769*		*0.001*		
B and C models (strong metric invariance)	3.71	3	0.295		0		
	*10.11*	*3*	*0.018*		*0.002*		
C and D models (strict metric invariance)	54.71	15	<0.001		0.008		
	*99.18*	*16*	*<0.001*		*0.007*		

Notes: the perception of boy-to-girl violence appears in the first line, normal font. The girl-to-boy violence appears in the second line, in italics.

**Table 6:** ANOVA results for the Questionnaire of Perception of Inter-Partner Violence from boy-to-girl and girl-to-boy.

Measurement	Inter-Subject Effect		
		Boy-To-Girl Violence	Girl-To-Boy Violence
Multiple Abuse	Sex	Girls: *N* = 198, *M* = 0.08, *SD* = 0.94	Girls: *N* = 198, *M* = 0.11, *SD* = 0.92
		Boys: *N* = 191, *M* = −0.08, *SD* = 1.02	Boys: *N* = 191, *M* = −0.11, *SD* = 1.02
		*F*_1,385_ = 3.28, *p* = 0.071, *η^2^_partial_* = 0.008	*F*_1,385_ = 6.40, *p* = 0.012, *η^2^_partial_* = 0.016
	Country of origin	Colombia: *N* = 117, *M* = 0.03, *SD* = 0.88	Colombia: *N* = 117, *M* = −0.07, *SD* = 0.96
		Spain: *N* = 272, *M* = −0.01, *SD* = 1.02	Spain: *N* = 272, *M* = 0.03, *SD* = 0.99
		*F*_1,385_ = 0.19, *p* = 0.666, *η^2^_partial_* = 0.000	*F*_1,385_ = 0.81, *p* = 0.368, *η^2^_partial_* = 0.002
	Interaction	Girls—Colombia: *N* = 59, *M* = 0.18, *SD* = 0.88	Girls—Colombia: *N* = 59, *M* = 0.13, *SD* = 0.95
		Girls—Spain: *N* = 139, *M* = 0.03, *SD* = 0.97	Girls—Spain: *N* = 139, *M* = 0.09, *SD* = 0.91
		Boys—Colombia: *N* = 58, *M* = −0.12, *SD* = 0.87	Boys—Colombia: *N* = 58, *M* = −0.27, *SD* = 0.94
		Boys—Spain: *N* = 133, *M* = −0.06, *SD* = 1.08	Boys—Spain: *N* = 133, *M* = −0.04, *SD* = 1.06
		*F*_1,385_ = 0.82, *p* = 0.367, *η^2^_partial_* = 0.002	*F*_1,385_ = 1.43, *p* = 0.232, *η^2^_partial_* = 0.004
	Levene’s test	*F*_3,385_ = 1.53, *p* = 0.206	*F*_3,385_ = 0.94, *p* = 0.424
Emotional Abuse	Sex	Girls: *N* = 198, *M* = 0.07, *SD* = 0.89	Girls: *N* = 198, *M* = −0.16, *SD* = 0.94
		Boys: *N* = 191, *M* = −0.03, *SD* = 1.00	Boys: *N* = 191, *M* = 0.07, *SD* = 0.97
		*F*_1,385_ = 2.17, *p* = 0.142, *η^2^_partial_* = 0.006	*F*_1,385_ = 0.95, *p* = 0.331, *η^2^_partial_* = 0.002
	Country of origin	Colombia: *N* = 117, *M* = 0.07, *SD* = 0.89	Colombia: *N* = 117, *M* = −0.07, *SD* = 0.96
		Spain: *N* = 272, *M* = −0.03, *SD* = 1.00	Spain: *N* = 272, *M* = 0.03, *SD* = 0.99
		*F*_1,385_ = 0.84, *p* = 0.359, *η^2^_partial_* = 0.002	*F*_1,385_ = 4.66, *p* = 0.031, *η^2^_partial_* = 0.012
	Interaction	Girls—Colombia: *N* = 59, *M* = 0.18, *SD* = 0.88	Girls—Colombia: *N* = 59, *M* = −0.04, *SD* = 0.91
		Girls—Spain: *N* = 139, *M* = 0.02, *SD* = 0.94	Girls—Spain: *N* = 139, *M* = 0.05, *SD* = 0.98
		Boys—Colombia: *N* = 58, *M* = −0.04, *SD* = 0.89	Boys—Colombia: *N* = 58, *M* = −0.28, *SD* = 0.97
		Boys—Spain: *N* = 133, *M* = −0.08, *SD* = 1.06	Boys—Spain: *N* = 133, *M* = 0.08, *SD* = 0.97
		*F*_1,385_ = 0.36, *p* = 0.548, *η^2^_partial_* = 0.001	*F*_1,385_ = 1.55, *p* = 0.214, *η^2^_partial_* = 0.004
	Levene’s test	*F*_3,385_ = 1.35, *p* = 0.257	*F*_3,385_ = 0.35, *p* = 0.787

Notes: Item values are summative standardized scores.

**Table 7:** Regression coefficients of the different logistic regression models.

Perception of Boy-to-Girl Violence in Each Country				
Predictor	*β*	Contrast Statistic	*e^β^*	*Odds*
Constant	0.889	*Wald*(1) = 6.18, *p* = 0.013	2.433	143.3% for Spain
Item 13	0.596	*Wald*(1) = 6.51, *p* = 0.011	1.816	81.6% for Spain
Item 9	−0.321	*Wald*(1) = 4.17, *p* = 0.041	0.725	27.5% for Colombia
Item 7	−0.88	*Wald*(1) = 19.44, *p* < 0.001	0.415	58.5% for Colombia
Item 2	0.481	*Wald*(1) = 7.02, *p* = 0.008	1.618	61.8% for Spain
**Perception of Girl-to-Boy Violence in Each Country**				
Constant	0.552	*Wald*(1) = 2.01, *p* = 0.156	1.686	68.6% for Spain
Item 14	−0.541	*Wald*(1) = 4.88, *p* = 0.027	0.582	41.8% for Colombia
Item 12	0.655	*Wald*(1) = 12.14, *p* < 0.001	1.926	92.6% for Spain
Item 7	−1.053	*Wald*(1) = 20.79, *p* < 0.001	0.349	65.1% for Colombia
Item 6	−0.563	*Wald*(1) = 4.45, *p* = 0.035	0.57	43.0% for Colombia
Item 5	0.804	*Wald*(1) = 9.01, *p* = 0.003	2.234	123.4% for Spain
Item 4	−0.357	*Wald*(1) = 3.93, *p* = 0.047	0.699	30.1% for Colombia
Item 2	1.292	*Wald*(1) = 34.35, *p* < 0.001	3.641	264.1% for Spain
**Perception of Boy-to-Girl Violence in Each Gender**				
Constant	0.333	*Wald*(1) = 0.979, *p* = 0.322	1.395	39.5% for boys
Item 10	0.355	*Wald*(1) = 4.17, *p* = 0.041	1.427	42.7% for boys
Item 8	−0.568	*Wald*(1) = 12.38, *p* < 0.001	0.567	43.3% for girls
**Perception of Girl-to-Boy Violence in Each Gender**				
Constant	0.525	*Wald*(1) = 3.64, *p* = 0.056	1.691	69.1% for boys
Item 8	−0.526	*Wald*(1) = 19.20, *p* < 0.001	0.591	40.9% for girls
Item 4	0.3	*Wald*(1) = 6.66, *p* = 0.010	1.35	35.0% for boys
